# Clinical characteristics and prognosis of patients with very severe acute hypertension visiting the emergency department

**DOI:** 10.1186/s40885-022-00208-3

**Published:** 2022-08-15

**Authors:** Hyun-Jin Kim, Byung Sik Kim, Jeong-Hun Shin

**Affiliations:** Division of Cardiology, Department of Internal Medicine, Hanyang University College of Medicine, Hanyang University Guri Hospital, 153 Gyeongchun-ro, Guri, Gyeonggi-do 11923 Republic of Korea

**Keywords:** Very severe acute hypertension, Emergency department, Hypertension-mediated organ damage, Mortality

## Abstract

**Background:**

Data regarding very severe acute hypertension, a serious problem in emergency departments (EDs), are scarce. We investigated the clinical characteristics, practice patterns, and long-term prognoses of patients presenting to the ED with very severe acute hypertension.

**Methods:**

Cross-sectional study data were obtained from a single regional emergency medical center, including patients aged ≥ 18 years who were admitted to the ED between January 2016 and December 2019 for very severe acute hypertension, which was defined as systolic blood pressure of > 220 mmHg and/or diastolic blood pressure of > 120 mmHg. The patients were classified into two groups based on the presence or absence of hypertension-mediated organ damage (HMOD).

**Results:**

Among 1,391 patients with very severe acute hypertension in the ED, half of the them (50.2%) had a previous medical history of hypertension, and 547 (39.3%) had acute HMOD. The overall 3-month, 1-year, and 3-year mortality rates were 5.2%, 11.9%, and 17.3%, respectively. In particular, patients with HMOD had a significantly higher mortality rate at each time point than those without HMOD. Among patients with HMOD, acute ischemic stroke was the most common (28.7%). Moreover, intravenous antihypertensive drugs were significantly more prescribed in patients with HMOD than in those without HMOD (79.0% vs. 22.2%, *P* < 0.001), but there were no differences in oral antihypertensive drugs between the two groups.

**Conclusions:**

Patients with very severe acute hypertension had poor long-term clinical prognoses. Clinicians should be continuously monitoring and providing appropriate treatment and close follow-up for patients with very severe acute hypertension.

**Supplementary Information:**

The online version contains supplementary material available at 10.1186/s40885-022-00208-3.

## Background

Hypertension is an important risk factor for cardiovascular and cerebrovascular events [1], and the number of patients with hypertension has steadily increased to more than 12 million in Korea [2]. Recently, although clinical trials and meta-analyses have strongly suggested intensive blood pressure (BP) lowering in patients with hypertension [3], approximately 1%–2% of patients eventually develop a severe acute increase in BP [4]. Hypertensive crisis or severe hypertension is defined as a BP elevation above 180 mmHg for systolic BP (SBP) and/or above 110–120 mmHg for diastolic blood pressure (DBP) according to the international practice guidelines for hypertension [5, 6]. Hypertensive emergency (HE) and hypertensive urgency, often referred to as hypertensive crisis, are distinguished by the presence or absence of new onset or worsening of acute organ damage [5–7]. According to our previous study, patients with severe acute hypertension accounted for 59 out of 1,000 emergency department (ED) visits at a single center in Korea, and their definition of severe acute hypertension was an SBP of ≥ 180 mmHg and/or a DBP of ≥ 100 mmHg [7]. However, a BP that is much higher than this threshold is often and is a life-threatening condition encountered in the EDs, which is highly associated with morbidity and mortality [8]. Immediate BP-lowering treatment is needed to prevent the progression of target organ damage in patients with severe acute BP elevation. Despite the clinical significance of severe acute BP elevation, epidemiologic studies and long-term outcomes in patients with very severe acute hypertension are scarce [6]. In this study, we investigated the clinical characteristics, practice patterns, and long-term prognosis of patients presenting to the ED with very severe acute hypertension.

## Method

### Study population and data collection

**Fig. 1:**
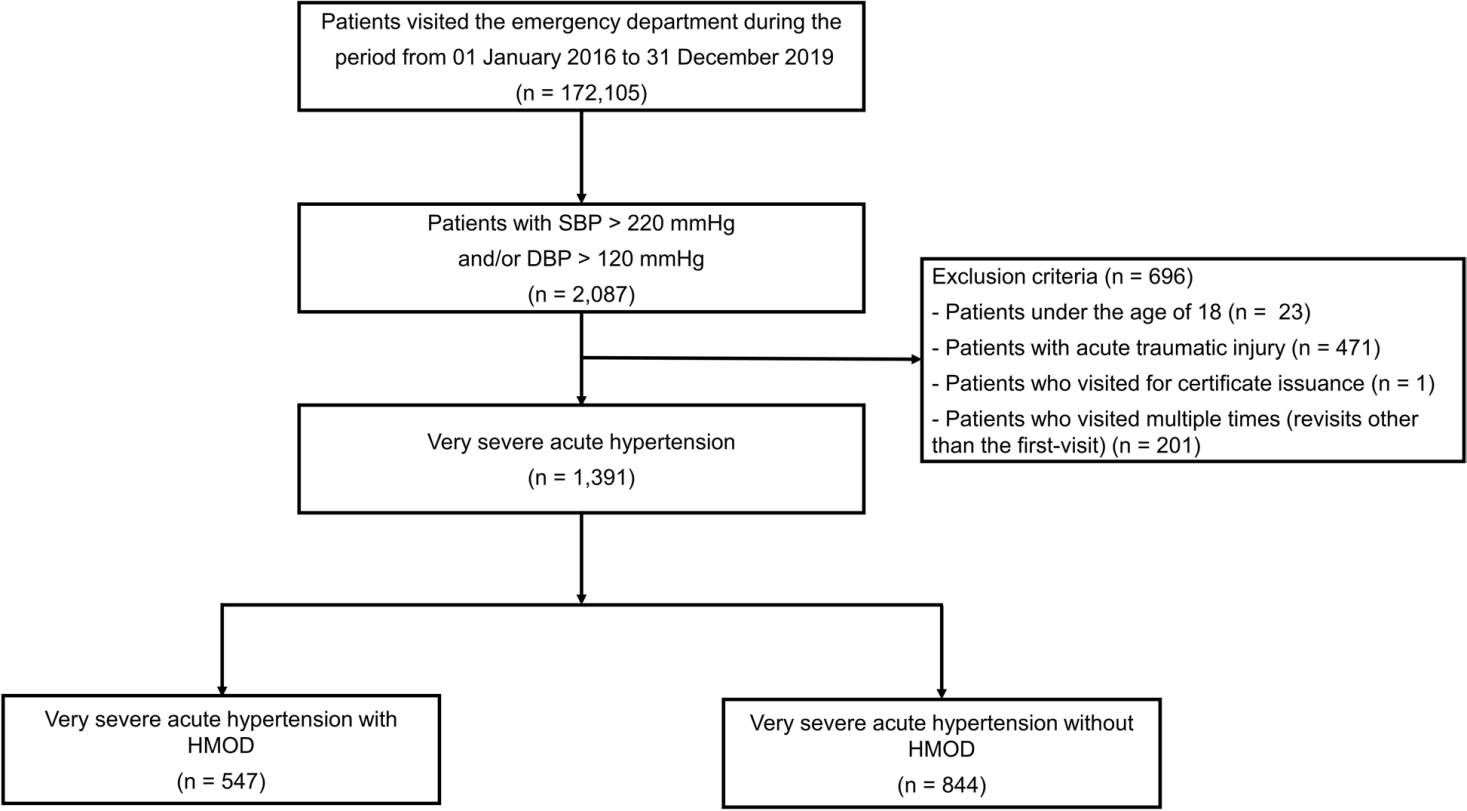
Study population

Cross-sectional study data were obtained from a single regional emergency medical center affiliated with an academic university hospital in Guri, Gyeonggi-do, Korea. The study design and primary results were published previously [7]. In brief, the medical records of 172,105 patients who visited the ED between January 2016 and December 2019 were reviewed; 2,087 patients who had an elevated initial SBP of > 220 mmHg and/or a DBP of > 120 mmHg were enrolled in this study (Fig. 1). Of these, patients who presented following acute trauma, for the certificates, or who visited the ED multiple times (revisits other than the first-visit) were excluded from this study. Finally, 1,391 patients with very severe acute hypertension were further classified based on the presence or absence of hypertension-mediated organ damage (HMOD). For this process, the National Emergency Department Information System (NEDIS) data were used to identify eligible patients who visited the ED. The system collects data, including initial vital signs and baseline clinical characteristics, and this information on all patients visiting the ED is automatically transmitted from each hospital to a central government server called the NEDIS.

The study was conducted in accordance with the Declaration of Helsinki and reviewed and approved by the Institutional Review Board of the researchers’ affiliated university hospital (GURI 2020–01-028), which waived the requirement for written informed consent.

### Data collection

Data were collected using electronic medical records by experienced data collectors under principal investigator supervision. The following demographic and clinical characteristics were extracted: age, sex, initial BP in the ED, and traditional cardiovascular risk factors, including a history of hypertension, diabetes, dyslipidemia, ischemic stroke, hemorrhagic stroke, coronary artery disease, peripheral artery disease, heart failure, chronic kidney disease, and end-stage renal disease and smoking and alcohol consumption status.

The following laboratory data were extracted: serum creatinine, estimated glomerular filtration rate (eGFR) (mL/min/1.73 m^2^), troponin I, B-type natriuretic peptide (BNP), D-dimer, and proteinuria in a dipstick urinalysis. In addition, the diagnostic test findings to determine HMOD, types of antihypertensive drugs for controlling BP in the ED, and principal diagnosis at discharge were obtained.

The primary outcome was the development of all-cause mortality during the 3-year follow-up (until March 2021). The incidence of mortality and its timing were extracted from the National Health Insurance Service in South Korea. The secondary outcomes were all-cause mortality within 1 month, 3 months, and 1 year; hospital admission; discharge in the ED; discharge against medical advice; and mortality in the ED.

### Definition 

In this study, very severe acute hypertension was defined as an initial SBP of > 220 mmHg and/or DBP of > 120 mmHg when visiting the ED. HMOD was defined as hypertensive encephalopathy, cerebral infarction, intracerebral hemorrhage, retinopathy, acute heart failure, acute coronary syndrome, acute renal failure, and aortic dissection [9]. Hypertensive encephalopathy was defined as a severe increase in BP associated with other unexplained lethargy, seizures, cortical blindness, and coma [9]. Cerebral infarction and intracerebral hemorrhage were defined based on neurologic symptoms and brain imaging in the ED. Fundus examination confirmed retinopathy based on the presence of flame-like hemorrhages, cotton wool spots, or papilledema. Proteinuria was defined as a dipstick urinalysis result of 1 + or greater [10]. The BP was measured in the ED above the brachial artery using an automated BP machine, Spot Vital Signs LXi (Welch Allyn, Skaneateles Falls, NY, USA).

### Statistical analysis

All categorical data are presented as frequencies and percentages, and data for continuous variables are expressed as means and standard deviations. Pearson’s chi-square test or Fisher’s exact test was used to compare categorical variables. Student’s *t*-test was used to compare continuous variables. The Kaplan–Meier survival analyses and log-rank tests were used to compare the cumulative mortality rates. Significance was defined as a *P* value of < 0.05. All analyses were performed using SPSS v26 (IBM Inc., Armonk, NY, USA).

## Results

### Clinical characteristics

Of the 172,105 patients who visited the ED during the study period, 1,391 (0.8%) had very severe acute hypertension, and 547 (39.3%) had HMOD. The patients’ baseline characteristics, including medical history, laboratory findings, and presenting symptoms, are presented in Table 1. The mean age of the total study population was 57.4 ± 17.2 years old, and 46.2% were women. Half of the patients (50.2%) had a previous medical history of hypertension. Of those who had hypertension previously, 358 (61.1%) were currently taking antihypertensive drugs. Blood tests (88.2%), chest radiography (84.5%), and electrocardiography (ECG) (79.7%) were performed in most patients with very severe acute hypertension, and brain imaging including brain computed tomography (CT) or magnetic resonance imaging (MRI) was performed in less than half (41.5%). Chest and abdominal CT, echocardiography, and fundoscopy were performed in a small number of patients. Patients with HMOD were significantly older and predominantly men compared with those without HMOD. In addition, patients with HMOD had significantly more traditional cardiovascular risk factors, including hypertension, diabetes, dyslipidemia, hemorrhagic stroke, coronary artery disease, heart failure, chronic kidney disease, end-stage renal disease, current smoking, and alcohol consumption than those without HMOD. Compared with patients without HMOD, those with HMOD presented with a significantly higher mean SBP (217.6 ± 29.0 vs. 198.2 ± 27.9 mmHg, *P* < 0.001). The DBP did not differ between the two groups. The mean eGFR was significantly lower in patients with HMOD than in those without HMOD, and proteinuria was more frequently observed in patients with HMOD. The mean BNP value was significantly higher in patients with HMOD, and cardiomegaly and congestion on chest radiography were significantly more observed in patients with HMOD than in those without HMOD. Abnormal findings on ECG, brain imaging, and fundoscopy were also more frequently observed in patients with HMOD.

**Table 1:** Baseline characteristics

	All patients (*n* = 1,391)	Patients with HMOD (*n* = 547)	Patients without HMOD (*n* = 844)	*P* value
Mean age, years (SD)	57.4 (17.2)	62.1 (15.3)	54.4 (17.7)	< 0.001
Female sex, N (%)	642 (46.2)	225 (41.1)	417 (49.4)	0.003
Medical history, N (%)				
Hypertension	674 (50.2)	326 (61.2)	348 (43.0)	< 0.001
Diabetes mellitus	306 (23.1)	149 (28.1)	157 (19.8)	< 0.001
Dyslipidemia	89 (6.8)	47 (8.9)	42 (5.4)	0.013
Ischemic stroke	80 (6.1)	37 (7.0)	43 (5.5)	0.267
Hemorrhagic stroke	45 (3.4)	25 (4.7)	20 (2.6)	0.035
Coronary artery disease^a^	95 (7.2)	60 (11.3)	35 (4.5)	< 0.001
Peripheral artery disease	17 (1.3)	6 (1.1)	11 (1.4)	0.666
Heart failure	61 (4.7)	51 (9.6)	10 (1.3)	< 0.001
Chronic kidney disease	108 (8.2)	62 (11.7)	46 (5.9)	< 0.001
End-stage renal disease	64 (4.9)	33 (6.2)	31 (4.0)	0.062
Social history, N (%)				
Cigarette smoking	294 (32.5)	162 (35.9)	132 (29.0)	0.042
Alcohol consumption	406 (43.7)	216 (47.5)	190 (40.0)	0.022
Triage vitals, mean (SD)				
SBP, mmHg	205.4 (29.7)	217.6 (29.0)	198.2 (27.9)	< 0.001
DBP, mmHg	128.1 (15.6)	128.3 (17.6)	128.1 (14.1)	0.814
Laboratory tests done, N (%)	1227 (88.2)	539 (98.5)	688 (81.5)	< 0.001
Mean serum creatinine, mg/dL (SD)	1.39 (1.9)	1.62 (2.2)	1.22 (1.7)	< 0.001
Mean eGFR, mL/min/1.73 m^2^ (SD)	79.5 (32.4)	71.4 (32.3)	85.7 (31.1)	< 0.001
Troponin I, ng/mL (SD)	0.249 (3.28)	0.437 (4.67)	0.080 (0.86)	0.126
BNP, pg/mL (SD)	519.9 (971.2)	677.1 (1051.1)	305.8 (806.2)	< 0.001
D-dimer, mg/L (SD)	804.8 (2546.0)	783.5 (2159.2)	830.5 (2982.6)	0.810
Urinary analysis done, N (%)	849 (61.0)	406 (74.2)	443 (52.5)	< 0.001
Proteinuria, N (%)^b^	329 (38.8)	181 (44.6)	148 (33.4)	< 0.001
Chest X-ray done, N (%)	1175 (84.5)	530 (96.9)	645 (76.4)	< 0.001
Cardiomegaly, N (%)	176 (14.7)	96 (18.0)	80 (12.0)	0.003
Congestion/fluid overload, N (%)	97 (8.1)	96 (18.0)	1 (0.2)	< 0.001
ECG done, N (%)	1109 (79.7)	525 (96.0)	584 (69.2)	< 0.001
LVH, N (%)	190 (17.2)	120 (22.9)	70 (12.0)	< 0.001
Myocardial ischemia, N (%)	78 (7.1)	58 (11.1)	20 (3.4)	< 0.001
Atrial fibrillation, N (%)	79 (7.1)	54 (10.3)	25 (4.3)	< 0.001
Brain CT or MR done, N (%)	577 (41.5)	328 (60.0)	249 (29.5)	< 0.001
Abnormal finding, N (%)	281 (42.8)	264 (75.9)	17 (5.5)	< 0.001
Chest and abdomen CT done, N (%)	144 (10.4)	55 (10.1)	89 (10.5)	0.769
Echocardiography done, N (%)	43 (3.1)	39 (7.1)	4 (0.5)	< 0.001
Fundoscopy done, N (%)	50 (3.6)	22 (4.0)	28 (3.3)	0.491
Abnormal finding, N (%)	18 (13.8)	13 (27.1)	5 (6.1)	< 0.001
Current antihypertensive drugs, N (%)^c^	358 (61.1)	186 (63.1)	172 (59.1)	0.327
Presenting symptoms, n (%)				
Chest pain	126 (9.1)	82 (15.0)	44 (5.2)	< 0.001
Dyspnea	201 (14.5)	118 (21.6)	83 (9.8)	< 0.001
Headache	110 (7.9)	37 (6.8)	73 (8.6)	0.203
Dizziness	135 (9.7)	32 (5.9)	103 (12.2)	< 0.001
Altered mental status	180 (12.9)	128 (23.4)	52 (6.2)	< 0.001
Focal neurologic deficit	239 (17.2)	187 (34.2)	52 (6.2)	< 0.001
Visual disturbance	22 (1.6)	12 (2.2)	10 (1.2)	0.141
Epistaxis	53 (3.8)	0 (0)	53 (6.3)	< 0.001

Data shown with percentages in parentheses represent numbers of participants, and other data represent mean values with standard deviation in parentheses; *BNP* B-type natriuretic peptide, *CT* computed tomography, *DBP* diastolic blood pressure, *eGFR* estimated glomerular filtration rate, *ECG* electrocardiogram, *HMOD* hypertension-mediated organ damage, *LVH* left ventricular hypertrophy, *MR* Magnetic resonance, *N* Number, *SBP* Systolic blood pressure, *SD* Standard deviation

^a^Composite of coronary artery disease, percutaneous coronary intervention, and coronary artery bypass graft

^b^Proteinuria is defined as a dipstick urinalysis result of 1 + or more

^c^The proportion of current antihypertensive drugs of those who had hypertension previously

### Antihypertensive therapy and BP changes in the ED

**Fig. 2:**
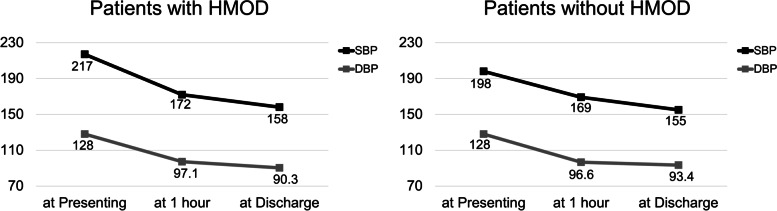
Blood pressure changes in the emergency department in patients with very severe acute hypertension according to hypertension-mediated organ damage

Among 1,391 patients with very severe acute hypertension, 619 (44.5%) received intravenous antihypertensive drugs, and 268 (19.3%) received oral antihypertensive drugs in the ED (Table 2). Intravenous nicardipine was the most frequently prescribed (32.6%), and intravenous nitroglycerine was the second most common (15.2%). Among oral antihypertensive drugs, calcium antagonists were the most commonly prescribed drugs. Moreover, intravenous antihypertensive drugs were significantly more prescribed in patients with HMOD than in those without HMOD (79.0% vs. 22.2%, *P* < 0.001), but there was no difference in oral antihypertensive drugs between the two groups. Figure 2 shows the BP changes within 1 h and at discharge in both groups. The BP was measured within 1 h in only 39.0% of all patients (59.2% of patients with HMOD and 25.9% of those without HMOD), and the BP at discharge was assessed in 64.6% of all patients (80.1% of patients with HMOD and 54.6% of those without HMOD). Initially, the mean SBP and DBP were higher in patients with HMOD than in those without HMOD. The BPs within 1 h and at discharge were also higher in patients with HMOD.

**Table 2:** Antihypertensive drugs used to control blood pressure for patient with very severe acute hypertension in ED

	All patients (*n* = 1,391)	Patients with HMOD (*n* = 547)	Patients without HMOD (*n* = 844)	*P* value
Oral antihypertensive drugs	268 (19.3)	106 (19.4)	162 (19.2)	0.932
Calcium antagonists^a^	170 (12.2)	38 (6.9)	132 (15.6)	< 0.001
Beta-blocker^b^	62 (4.5)	31 (5.7)	31 (3.7)	0.078
Renin-angiotensin system inhibitor^c^	22 (1.6)	13 (2.4)	9 (1.1)	0.056
Nitroglycerin	73 (5.2)	54 (9.9)	19 (2.3)	< 0.001
Intravenous antihypertensive drugs	619 (44.5)	432 (79.0)	187 (22.2)	< 0.001
Nicardipine	453 (32.6)	297 (54.3)	156 (18.5)	< 0.001
Labetalol	35 (2.5)	28 (5.1)	7 (0.8)	< 0.001
Esmolol	14 (1.0)	12 (2.2)	2 (0.2)	< 0.001
Nitroglycerin	211 (15.2)	173 (31.6)	38 (4.5)	< 0.001
Cessation due to iatrogenic hypotension	71 (5.1)	41 (7.5)	30 (3.6)	0.048

*HMOD* hypertension-mediated organ damage

^a^Calcium antagonists included amlodipine and nifedipine

^b^Beta-blocker included carvedilol, nebivolol, propranolol, atenolol, and bisoprolol

^c^Renin-angiotensin system inhibitor included perindopril, candesartan, losartan, and fimasartan

### Patterns of HMOD and principal diagnosis at discharge

Among the 547 patients with HMOD, acute ischemic stroke was the most common (28.7%), followed by acute heart failure, intracerebral hemorrhage, acute coronary syndrome, acute kidney injury, subarachnoid hemorrhage, hypertensive encephalopathy, hypertensive retinopathy, and aortic dissection (Supplementary Table S1). Among the 1,391 patients, neurologic disorders, including ischemic stroke, hemorrhagic stroke, and seizures, were the most common primary diagnoses (29.3%) at discharge (Supplementary Table S2), followed by cardiovascular disorders (25.6%).

### Clinical outcomes during the follow-up period

Overall, 755 (54.3%) patients were admitted, 487 (35.0%) were discharged, 146 (10.5%) were discharged against medical advice, and 3 (0.2%) died in the ED (Table 3). Most of the patients with HMOD (91.4%) were admitted to the general ward or intensive care units (ICU), and patients without HMOD were significantly more commonly discharged from the ED than those with HMOD (56.4% vs. 2.0%, *P* < 0.001).

**Table 3:** Clinical outcome according to follow-up duration

	All patients (*n* = 1,391)	Patients with HMOD (*n* = 547)	Patients without HMOD (*n* = 844)	*P* value
Mortality rate within 1-month	72 (5.2)	50 (9.1)	22 (2.6)	< 0.001
Mortality rate within 3-months	106 (7.6)	71 (13.0)	35 (4.1)	< 0.001
Mortality rate within 1-year	166 (11.9)	99 (18.1)	67 (7.9)	< 0.001
Mortality rate within 3-years	241 (17.3)	136 (24.9)	105 (12.4)	< 0.001
Admission	755 (54.3)	500 (91.4)	255 (30.2)	< 0.001
Discharge at ED	487 (35.0)	11 (2.0)	476 (56.4)	< 0.001
Discharge against medical advice	146 (10.5)	35 (6.4)	111 (13.2)	< 0.001
Death in the emergency department	3 (0.2)	1 (0.2)	2 (0.2)	0.832

*HMOD* hypertension-mediated organ damage, *ED* emergent department

**Fig. 3:**
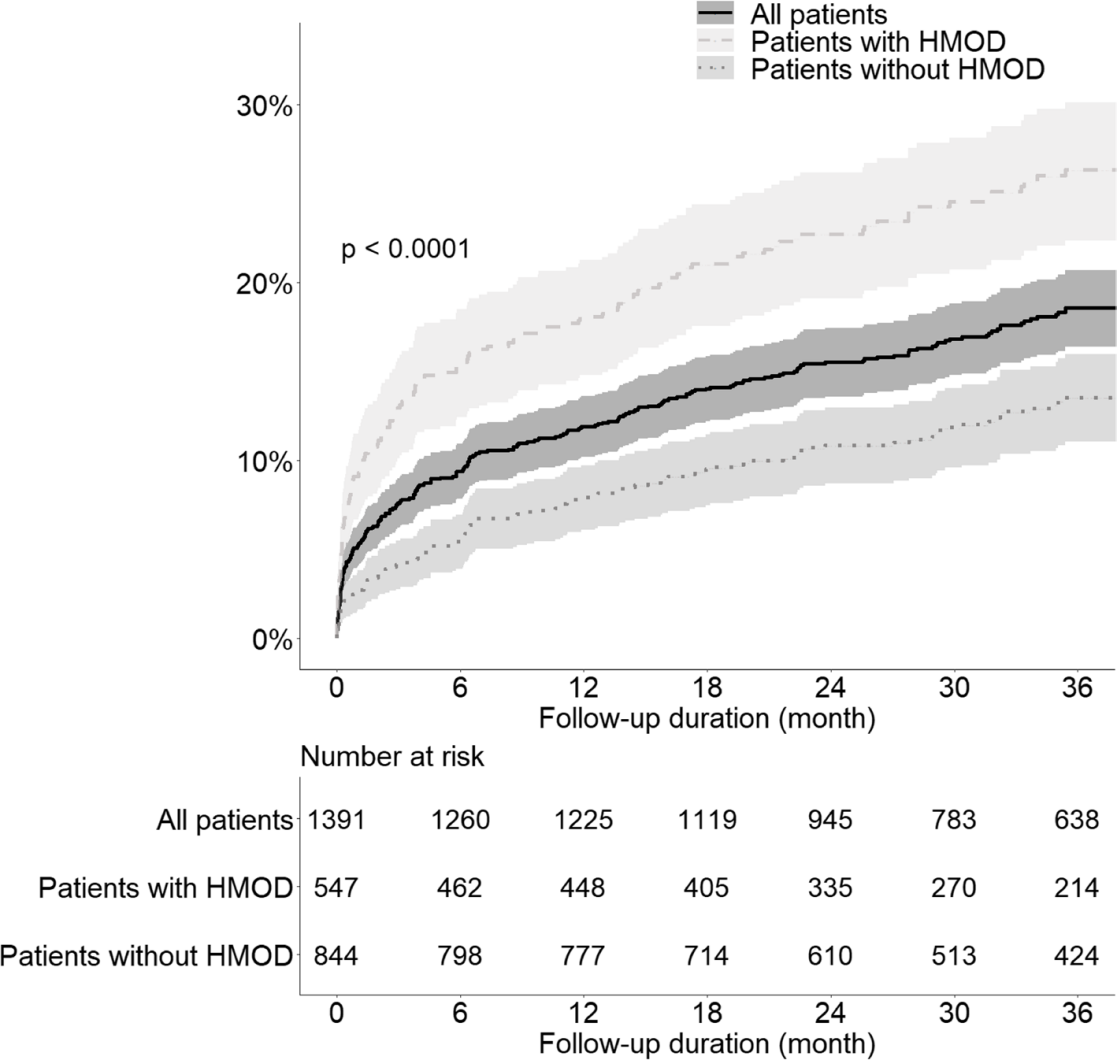
Cumulative mortality rate in all patients and in those stratified according to the presence of hypertension-mediated organ damage

Among 1,391 patients, 214 (17.3%) died within 3 years, and the mortality rates increased over time from 1 month to 3 years. In particular, patients with HMOD had a significantly higher mortality rate at each time point than those without HMOD. Figure 3 shows the cumulative mortality rate in all patients according to the presence of HMOD using the Kaplan–Meier curves. Patients with HMOD had a significantly higher cumulative mortality rate than those without HMOD (log-rank *P* < 0.001).

Of all patients, except 72 who died within a month, 1,009 (76.5%) of those discharged from the ED or after hospitalization attended their follow-up appointment (85.9% of patients with HMOD and 70.8% of those without HMOD). Among these patients, especially in patients with HMOD, the 1-year mortality rate was significantly higher in patients who were lost to follow-up than in those who were kept on follow-up appointments (20.0% vs. 8.2%, *P* = 0.002) (Supplementary Table S3).

## Discussion

Based on the results of our study, the mortality rate within 3 years of patients who visited the ED for very severe acute hypertension was 17.3%, and especially, those with HMOD had worse prognosis. Although half of the patients (50.2%) were aware of their history of hypertension in this study, only 61.1% of them were taking antihypertensive drugs, suggesting that uncontrolled hypertension exists in patients receiving treatment as well as in untreated patients. Patients with very severe acute hypertension were most often diagnosed with neurologic disorders when discharged, and ischemic stroke was the most common among patients with HMOD.

Severe acute BP elevation is a prevalent and significant problem in patients visiting the ED. Although the BP criteria for severe hypertension follow the international practice guidelines for hypertension, there are no established criteria or definitions for BP that increases to a level that is significantly higher than that observed in severe hypertension [5, 6]. In addition, there are recommendations only for specific conditions in the treatment of patients presenting with very severe acute hypertension. In the European Society of Cardiology and European Society of Hypertension guidelines, for patients with a markedly elevated SBP or DBP (SBP of ≥ 220 mmHg, DBP of ≥ 120 mmHg), BP-lowering management is only mentioned in the presence of the acute phase of hemorrhagic and ischemic stroke [6]. Similarly, in the American College of Cardiology/American Heart Association guidelines, recommendations about BP-lowering management for patients with a markedly elevated BP of SBP higher than 220 mmHg are only described in the cerebrovascular disease part [5]. Acute intracerebral hemorrhage is common in patients with very severe acute hypertension (SBP of ≥ 220 mmHg and/or DBP of ≥ 120 mmHg), and these patients have an increased risk of death as well as a worse prognosis for neurologic recovery [11, 12]. However, few studies have evaluated patients visiting the ED for very severe acute hypertension regardless of the specific target organ damage, and there are few reports on the incidence rates, treatment status, and long-term prognosis. Our study showed that 0.8% (1,391 of 172,105) of patients who visited the ED during the study period had very severe acute hypertension and 60.7% (844 of 1,391) of patients with very severe acute hypertension had no HMOD. To the best of our knowledge, this study is the first to reveal antihypertensive drugs and clinical prognosis for very severe acute hypertension regardless of HMOD, which is rare but critical in the ED. In a previous study, Preston et al. [13] showed that 147 patients presenting to the ED at a single center with an SBP of ≥ 220 mmHg or DBP of ≥ 120 mmHg were evaluated for prognosis including repeat visits, target organ events, and hospitalizations for 2 years. They showed an increased risk of repeat visits (*n* = 389), new target organ events (*n* = 99), and hospitalizations (*n* = 76) in patients with very severe acute hypertension. While they did not reveal mortality data, we presented 1-month, 3-month, 1-year, and 3-year mortality data for a larger number of 1,391 patients and found that patients with very severe acute hypertension had a poor prognosis and high all-cause mortality rate within 3 years. Recently, we reported that the 1- and 3-year mortality rates for patients with severe acute hypertension were 8.8% and 13.9%, respectively [7]. In comparison, the 1- and 3-year mortality rates for patients with very severe acute hypertension in the present study were higher at 11.9% and 17.3%, respectively. We also showed the distribution of HMOD accompanied by very severe acute hypertension that acute ischemic stroke accounted for the most frequent HMOD, but acute heart failure, intracerebral hemorrhage, and acute coronary syndrome were also important accompanying organ damage. In addition, since neurologic and cardiovascular disorders account for most of the principal diagnoses at discharge, we suggest that the concept of a marked increase in the BP due to these causes should be considered when patients with very severe acute hypertension are encountered.

Since the guidelines recommend intravenous antihypertensive drugs for the treatment of BP-lowering in patients with HE [5, 6], intravenous antihypertensive drugs were prescribed significantly more for patients with very severe acute hypertension with HMOD in our study. Among the intravenous antihypertensive drugs recommended in the guidelines, nicardipine was administered the most, followed by nitroglycerine. Based on the results of our study, in addition to the worse clinical prognosis of very severe acute hypertension, the HMOD group had a significantly worse prognosis; therefore, more aggressive BP-lowering treatment would be needed than that in the non-HMOD group. Generally, it is recommended that patients with HMOD be treated in an ICU for close monitoring and reduction of the BP with parenteral antihypertensive drugs [5]. However, in our study, 8.4% of the patients with HMOD were discharged from the ED, and most of them were discharged against medical advice. Since the rate of follow-up loss among patients who were discharged from the ED was relatively high and the follow-up loss was associated with high mortality, the importance of hospitalization in patients with HMOD should be emphasized.

To our knowledge, we are the first to show the long-term natural course, including mortality, of patients who visit the ED due to acutely elevated blood pressure. Especially, a higher BP criterion (SBP of > 220 mmHg and/or a DBP of > 120 mmHg) could at least partially ameliorate the effect of typically transient BP elevations in acute ED settings. Very severe acute hypertension is associated with adverse outcomes and high utilization of medical services. In this context, understanding the natural processes, including all-cause mortality, in patients with very severe acute hypertension is pivotal in planning the basis for improving healthcare and prognosis.

## Limitations

This study has some limitations. First, this was a retrospective study. There were missing data on variables highly related to very severe acute hypertension, such as alcohol consumption, smoking status, and drug history. We only examined the current status of antihypertensive drug usage but did not investigate its status following discharge. Second, this study was a single-center experience, which may not be representative of the entire population. However, the number of study patients was large, and meaningful results were derived from this. Third, the follow-up measurement of the BP in the ED was insufficient, especially for the patients in the non-HMOD group; the subsequent BP data were insufficient because the BP was not significantly measured after 1 h and at discharge. In particular, if there were data on the time when the target BP was reached, it would have been possible to quantitatively explain the outcomes. Further research regarding the clinical outcomes and presence of HMOD according to the time taken to control BP in patients with acute severe hypertension who visited the ED is needed. Finally, our study was unable to present cardiovascular mortality because there were no data available on the cause of death for the National Health Insurance Service. However, the data provided by the National Health Insurance Service on all-cause mortality and the date of death are accurate and reliable.

## Conclusions

An SBP of > 220 mmHg and/or DBP of > 120 mmHg (very severe acute hypertension) is frequently accompanied by neurologic disorders, such as ischemic stroke, and patients with very severe acute hypertension have poor long-term clinical prognosis. In particular, both HMOD and non-HMOD groups had high all-cause mortality rate, although a significant difference in mortality rates was noted between the two groups. Clinicians should closely monitor and actively manage patients with very severe acute hypertension in the ED.

## Supplementary Information

**Additional file 1:** **Supplementary Table 1. **Distribution of acute hypertension mediated-organ damage. **Supplementary Table 2. **Principaldiagnosis at discharge of all patients. **Supplementary Table 3**. Mortality rates at 3 monthsand 1 year according to follow-up visits.

## Data Availability

The datasets used and/or analyzed during the current study are available from the corresponding author on reasonable request.

## Abbreviations

BNP: B-type natriuretic peptide
BP: Blood pressure
CT: Computed tomography
DBP: Diastolic blood pressure
ECG: Electrocardiography
ED: Emergency department
eGFR: Estimated glomerular filtration rate
HE: Hypertensive emergency
HMOD: Hypertension-mediated organ damage
ICU: Intensive care units
MRI: Magnetic resonance imaging
SBP: Systolic blood pressure
