# Supplementary material for: Clinical characteristics and prognosis of patients with very severe acute hypertension visiting the emergency department
Source: Clin Hypertens. 2022 Aug 15;28:23. doi: 10.1186/s40885-022-00208-3 (PMC9377086; doi:10.1186/s40885-022-00208-3)
Supplement: Supplementary file 1 — Additional file 1: Supplementary Table 1. Distribution of acute hypertension mediated-organ damage. Supplementary Table 2. Principaldiagnosis at discharge of all patients. Supplementary Table 3. Mortality rates at 3 monthsand 1 year according to follow-up visits. [file 40885_2022_208_MOESM1_ESM.docx]

**Supplementary Table 1.** Distribution of acute hypertension mediated-organ damage

| **Very severe hypertension with HMOD** | **N = 547** |
| --- | --- |
| Acute ischemic stroke | 157 (28.7) |
| Acute heart failure | 131 (23.9) |
| Intracerebral hemorrhage | 118 (21.6) |
| Acute coronary syndrome | 92 (16.8) |
| Acute kidney injury | 62 (11.3) |
| Subarachnoid hemorrhage | 32 (5.9) |
| Hypertensive encephalopathy | 16 (2.9) |
| Hypertensive retinopathy | 22 (4.0) |
| Aortic dissection | 12 (2.2) |

Data shown with percentages in parentheses represent numbers of participants, HMOD; hypertension-mediated organ damage

**Supplementary Table 2.** Principal diagnosis at discharge of all patients

|  | All patients (n =1,391) |
| --- | --- |
| Neurologic disorders | 407 (29.3) |
| Ischemic stroke | 142 (10.2) |
| Hemorrhagic stroke | 141 (10.1) |
| Seizures | 26 (1.9) |
| Cardiovascular disorders | 356 (25.6) |
| Acute heart failure | 126 (9.1) |
| Angina pectoris | 56 (4.0) |
| Acute myocardial infarction | 55 (4.0) |
| Gastrointestinal disorders | 137 (9.8) |
| Gastroenteritis | 80 (5.8) |
| Gastrointestinal bleeding | 11 (0.8) |
| Pancreatitis | 9 (0.6) |
| Infectious diseases | 103 (7.4) |
| Lower respiratory tract infection | 24 (1.7) |
| Upper respiratory tract infection | 23 (1.7) |
| Urinary tract infection | 23 (1.7) |
| Kidney diseases | 35 (2.5) |
| Acute kidney injury | 25 (1.8) |
| Progression of chronic kidney disease | 7 (0.5) |
| Complication of end-stage renal disease | 2 (0.1) |
| Respiratory disorders | 30 (2.2) |
| Asthma | 16 (1.2) |
| Chronic obstructive pulmonary disease | 6 (0.4) |
| Pneumothorax | 6 (0.4) |
| Malignancies | 13 (0.9) |
| Others | 310 (22.3) |
| Urinary tract stones | 45 (3.2) |
| Epistaxis | 41 (2.9) |
| Benign paroxysmal positional vertigo | 24 (1.7) |

Data shown with percentages in parentheses represent numbers of participants.

| **Supplementary Table 3**. Mortality rates at 3 months and 1 year according to follow-up visits. | | | | | | | |  | | | |
| --- | --- | --- | --- | --- | --- | --- | --- | --- | --- | --- | --- |
|  | All patients  (n = 1,319) | | | Patients with HMOD  (n = 497) | | | Patients without HMOD  (n = 822) | | | |  |
|  | Follow-up (+)  (n = 1,009) | Follow-up (-)  (n = 310) | *p*-value | Follow-up (+)  (n = 427) | Follow-up (-)  (n = 70) | *p*-value | Follow-up (+)  (n = 582) | | Follow-up (-)  (n = 240) | *p*-value |  |
| Mortality rate within 3-months | 21 (2.1) | 48 (4.2) | 0.040 | 15 (3.5) | 6 (8.6) | 0.099 | 6 (1.0) | | 7 (2.9) | 0.064 |  |
| Mortality rate within 1-year | 68 (6.7) | 26 (8.4) | 0.324 | 35 (8.2) | 14 (20.0) | 0.002 | 33 (5.7) | | 12 (5.0) | 0.701 |  |

Data are presented as n (%). HMOD, hypertension-mediated organ damage
